# Measuring Health-Related Quality of Life in Vietnamese Patients After Kidney Transplantation

**DOI:** 10.3389/fsurg.2021.646629

**Published:** 2021-08-17

**Authors:** Le Nguyen Vu, Nguyen Quang Nghia, Tran Minh Tuan, Tran Ha Phuong, Hoang-Long Vo, Khai Ninh Viet, Tran Binh Giang

**Affiliations:** ^1^Organ Transplantation Center, Viet Duc University Hospital, Hanoi, Vietnam; ^2^Institute for Preventive Medicine and Public Health, Hanoi Medical University, Hanoi, Vietnam; ^3^Department of Scientific Research and International Cooperation, Hanoi Medical University Hospital, Hanoi, Vietnam

**Keywords:** chronic kidney disease, kidney transplantation, quality of life, measurement, Vietnam

## Abstract

**Objectives:** To consider that the health-related quality of life (HRQOL) has become an inherent part of the patient outcomes in the care and treatment after kidney transplantation (KT). This study aimed to measure HRQOL among a representative sample size of patients after KT by using both the Short Form 36 (SF-36) and the Kidney Disease Quality of Life 36 (KDQOL-36).

**Methods and Results:** Data of this cross-sectional design were collected in the Organ Transplant Center, Viet Duc University Hospital (Hanoi, Vietnam) from January 2020 to March 2020 and included the patients aged 18 years or over after KT at 6 months, 1 year, and 3 years postoperatively. HRQOL was evaluated through face-to-face interviews by means of the SF-36 and KDQOL-36 measurement tools. According to the SF-36, the overall mean score of HRQOL was 69.13 ± 15.55 and the two domains were the highest scores of “Mental Health” (81.23 ± 14.28) and “General Health” (80.06 ± 14.81). When measuring with the KDQOL-36, the overall mean score was 68.67 ± 13.75 and was the highest in the domain “Symptoms and Problems of Kidney Disease” (87.06 ± 16.00). Both instruments had good reliability for those after KT. The reliability of the SF-36 was high with Cronbach's coefficients α = 0.90. There were positive relationships between the dimensions measured by the KDQOL-36 and SF-36 (correlation coefficient: 0.03–0.69). Similarly, the domains of the SF-36 also had positive correlations with the KDQOL-36 (correlation coefficient: 0.18–0.51). The correlation coefficient between overall HRQOL scores of the SF-36 and KDQOL-36 was 0.62, indicating a strong correlation between the SF-36 and KDQOL-36.

**Conclusions:** There were slight fluctuations in the HRQOL score in domains in the 3-year follow-up stages, suggesting not having clear change. The mean SF-36 score was consistent with the mean KDQOL-36 score. High reliability and strong correlation were found between two instruments of the SF-36 and KDQOL-36. This study provides the reliability and constructs validity in the combination of two sets of the SF-36 and KDQOL-36 scales for the assessment of HRQOL among post-KT patients, thereby assisting physicians and health professionals in the clinical decision-making, assessment of therapeutic efficacy, and understanding of treatment risk.

## Introduction

Health-related quality of life (HRQOL), a well-established aspect of health and general wellbeing, can be measured with a variety of instruments. In medicine, disease-specific HRQOL measures are designed to well reflect the HRQOL aspects of a particular disease (1). For chronic illness, physicians have often paid attention to HRQOL to measure the disease effects in their patients to better understand the impact level of an illness on the day-to-day life of a person. Chronic kidney disease (CKD), an important public health issue with the global increase, describes the gradual loss of kidney function in patients (2). CKD, in general, and end-stage kidney disease (ESKD), in particular, often lead to both physical and emotional symptoms that adversely impact the health and daily routine of patients (3). A large systematic study recently indicated that people with CKD have decreased quality of life and poorer socioeconomic circumstances as CKD progresses (2). Fortunately, the development and perfection of kidney replacement therapy (in the form of dialysis or kidney transplantation) have significantly improved survival rates in patients with ESKD, and HRQOL improves dramatically after the successful renal transplantation compared to patients maintained on the dialysis treatment (4). As the clinical practices have varied widely across the world with the increasing demands in many aspects, there needs to be an increased interest in the assessment of mental health aspects in patients with ESKD after kidney transplantation (KT).

In the context of the culture and value systems in each institution, routine use of HRQOL assessment may be useful in monitoring clinical transplant practice, informing treatment decision-making, and/or aiding in the allocation of transplant healthcare resources. Although the HRQOL advantages of renal transplantation are well established (5–7), large differences in quality of life are often observed depending on specific transplant cohorts. Importantly, most changes in the data in HRQOL have been reported from developed countries (3). Vietnam, a lower middle-income country, experiences rapid economic growth over the last decade with increasing social and healthcare outcomes (8). The current status posed a growing demand for end-stage renal failure management and KT in Vietnam (8). KT is still an emerging and challenging area in Vietnam, and insights on the aspects of HRQOL relevant to KT are needed to propose a suitable management strategy for post-KT patients. This study represents our largest effort at one institution in Vietnam to measure HRQOL among a representative sample size of patients after KT.

## Methods

### Study Design, Settings, and Participants

This was a cross-sectional study carried out in the Organ Transplant Center, Viet Duc University Hospital (Hanoi, Vietnam) from January 2020 to March 2020. We included the patients who were monitored after KT at our institution at 6 months, 1 year, and 3 years postoperatively. We excluded the patients (i) suffered from kidney transplant failure after 3-month transplantation and must return to dialysis; (ii) died after KT; and (iii) experienced KT at our institution but post-KT follow-up treatment at other institutions. A total of 153 patients were eligible for the final analysis.

### Data Collection

Written informed consent was obtained from participants prior to data collection. Data were collected by face-to-face interviews. Interviewers were the trained nurses at our institution. During the interview, HRQOL was assessed in studying patients according to the Short Form 36 (SF-36) and the Kidney Disease Quality of Life 36 (KDQOL-36).

### Health-Related Quality of Life

#### SF-36

The SF-36 is a generic health questionnaire measuring eight health domains, such as Physical Functioning (PF), Limitations in Daily Role Functioning due to Physical Problems (RP), Bodily Pain (BP), General Health (GH), Vitality (VT), Social Functioning (SF), Limitations in Daily Role Functioning due to Emotional Problems (RE), Mental Health (MH), and an item asking respondents about health change over the last year. Scores for each domain can range from 0 to 100, higher scores indicating a better health state. Scores on each scale were calculated based on the “half item rule.” Two summary scores were also calculated from the eight domains: the Physical Component Summary (PCS) and the Mental Health Component Summary (MCS) (9, 10).

#### Kidney Disease Quality of Life 36

The KDQOL-36 contains five subscales: Physical Component Summary (PCS), Mental Component Summary (MCS), Burden of Kidney Disease (BKD), Symptoms and Problems of Kidney Disease (SPKD), and Effects of Kidney Disease (EKD).

The KDQOL-36 has 36 items including the SF-12 version 1 (12 items total) and three kidney-specific scales (24 items total). The SF-12 yields the PCS and MCS, both of which are scored on a T-score metric. The three kidney-targeted scales assess BKD, SPKD, and EKD. The Burden scale has four items (e.g., “My kidney disease interferes too much with my life”) that are prompted with the context “How true or false is each of the following statements?” and have five response options that range from “Definitely true” to “Definitely false.” The Symptoms/Problems scale has 12 items, each representing a symptom or side effect of kidney disease (e.g., “Washed out or drained?”) that are given the context “During the past 4 weeks, to what extent were you bothered by each of the following?” and have five response options ranging from “Not at all bothered” to “Extremely bothered.” The Effects scale has eight items (e.g., “Your ability to work around the house?”) with the context “How much does kidney disease bother you in each of the following areas?” and the same response options as the Symptoms/Problems subscale. Each of these scales is scored by transforming all items to a 0–100 possible range and averaging across the items on each scale to create scale scores. The KDQOL-36 items are all scaled so that higher scores indicate better HRQOL. The previously published norms (unadjusted means) for these scales are Burden = 41, Symptoms/Problems = 71, and Effects = 63. Although the KDQOL-36 has been translated into more than 25 different languages, a valid Vietnamese translation version was published by *RAND Health Care* (11).

#### Data Analysis

Data were sorted, cleaned, coded, and entered into Epidata 3.1 and analyzed using Statistical Package for Stata 13.1 software. Socioeconomic characteristics were summarized using descriptive statistics. Quantitative data were expressed as mean, SD, and interquartile range (IQR), whereas categorical data were expressed as frequency and percentage. Then, we calculated mean, SD, and Cronbach's alpha values of eight domains (PF, RP, BP, GH, VT, SF, RE, and MH) for the SF-36 and of five domains (PCS, MCS, BKD, SPKD, and EKD) for the KDQOL-36. The degree of association in overall HRQOL between the SF-36 and KDQOL-36 was measured by Pearson's correlation coefficient and denoted by r. Internal consistency reliability was evaluated using Cronbach's alpha coefficient calculated separately for each subscale. Coefficient alpha of 0.70 or greater is generally considered to be acceptable (12).

## Results

### Socioeconomic Characteristics of the Patients After Kidney Transplantation

Out of the 153 KT patients interviewed, 114 (74.51%) were men. The age of patients ranged from 19 to 70 years, with an average age of 39.48 ± 10.76 years. They were predominantly living in urban areas (62.75%) and were married (69.93%). Out of these, 45.75% of patients were officers, 29.41% were freelancers, 11.11% were farmers, 7.19% were students, and 6.54% were retired. Most patients had the education of upper secondary or higher (93.46%) (Table 1).

**Table 1:** Patient characteristics.

**Characteristics (*n* = 149)**	**Number (%)**
Age (years)	
Mean (SD)	39.48 ± 10.76
IQR	19–70
Gender	
Male	114 (74.51)
Female	39 (25.49)
Living area	
Rural	57 (37.25)
Urban	96 (62.75)
Comorbidity	
Diabetes	11 (7.19)
Hypertension	34 (23.78)
Others	108 (69.03)
Occupation	
Officer	70 (45.75)
Farmer	17 (11.11)
Freelancer	45 (29.41)
Student	11 (7.19)
Retirer	10 (6.54)
Education	
Lower secondary or lower	10 (6.54)
Upper secondary	44 (28.76)
University or higher	99 (64.71)
Marital status	
Single	39 (25.49)
Married	107 (69.93)
Widowed/divorced	7 (4.58)
Time on dialysis (months)	
Mean (SD)	38.45 (88.33)
IQR	0–961

Of the post-KT patients, 60.78% were followed up for 1 year or more and 39.22% were followed up under 1 year. The mean time on dialysis was 38.45 ± 88.33 months. The most common comorbidities were hypertension (23.78%) and diabetes (7.19%). After KT, most patients reported facing an economic burden and 50.30% of them were severely affected (Table 1).

### Health-Related Quality of Life Measured by Use of the SF-36 and KDQOL-36

**Figure 1:**
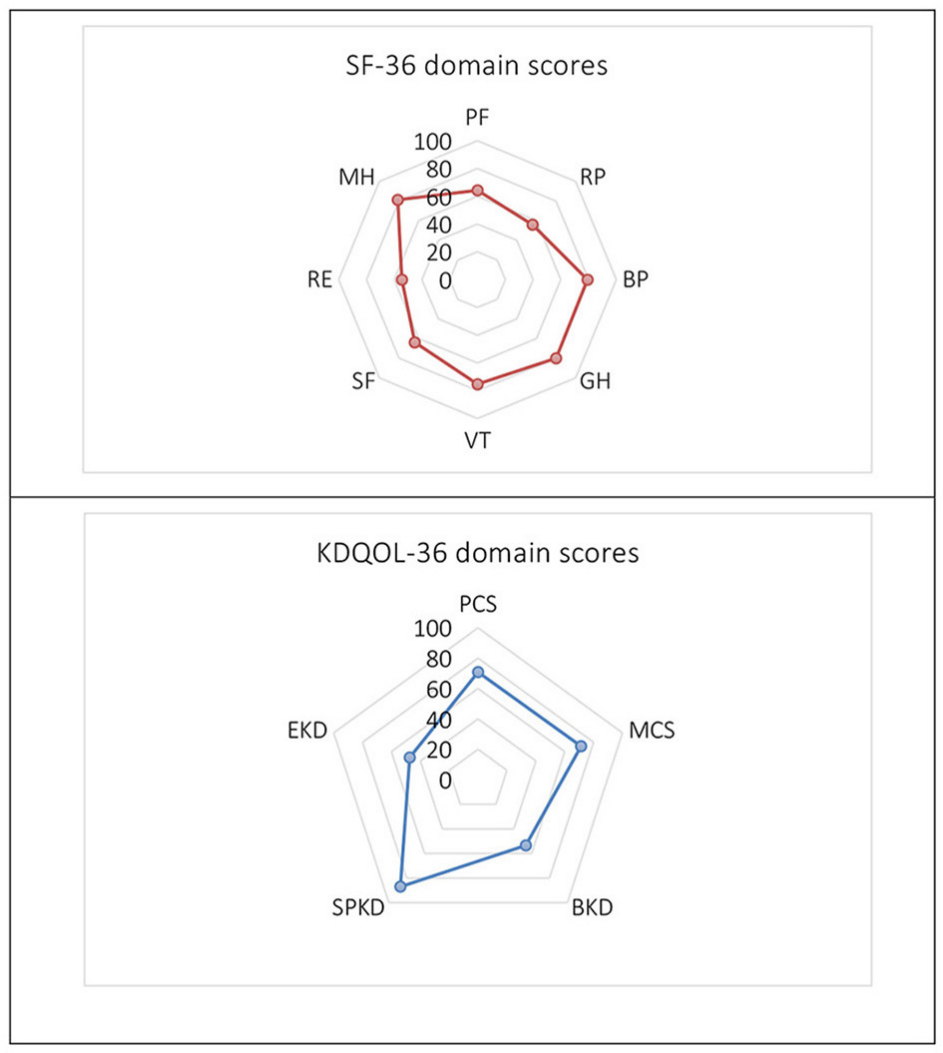
The Short Form 36 (SF-36) and Kidney Disease Quality of Life 36 (KDQOL-36) domain scores. PF, Physical Functioning; RP, Limitations in Daily Role Functioning due to Physical Problems; BP, Bodily Pain; GH, General Health; VT, Vitality; SF, Social Functioning; RE, Limitations in Daily Role Functioning due to Emotional Problems; MH, Mental Health; PCS, Physical Component Summary; MCS, Mental Component Summary; BKD, Burden of Kidney Disease; SPKD, Symptoms and Problems of Kidney Disease; EKD, Effects of Kidney Disease.

The distribution of domain scores by the SF-36 and KDQOL-36 scales was shown in Figure 1. We also examined the changes in the HRQOL scores in the domains at below 6 months, 6 months−1 year, 1–3 years, and above 3 years after transplantation (Table 2). According to the SF-36, the overall mean score of HRQOL was 69.13 ± 15.55 and the two dimensions were the highest scores of MH (81.23 ± 14.28) and GH (80.06 ± 14.81). Two dimensions of RP and RE were observed with the lowest mean scores of 56.04 ± 42.63 and 54.47 ± 43.38, respectively. In particular, the score increases gradually in BP, SF, and RE over time and their highest scores were observed after 3-year transplantation, at 84.91 ± 16.72, 66.38 ± 17.39, and 57.47 ± 42.63, respectively. The remaining dimensions of HRQOL commonly had the highest scores in the short-term duration within 6–12 months (Table 2).

**Table 2:** Component scores of the Short Form 36 (SF-36) and Kidney Disease Quality of Life 36 (KDQOL-36) by post-kidney transplant stages and Cronbach's alpha values of the domains.

	**Below 6 months (*n* = 39)**	**6 months to 1 year (*n* = 21)**	**1 year to 3 years (*n* = 64)**	**Above 3 years (*n* = 29)**	**Overall (*n* = 153)**	**Cronbach's α**
**SF-36**						
PF	61.15 ± 22.81	**67.86 ± 17.79**	66.80 ± 18.86	60.34 ± 22.56	64.28 ± 20.56	0.84
RP	41.67 ± 41.49	61.90 ± 45.15	**62.50 ± 40.58**	56.90 ± 44.27	56.04 ± 42.63	0.88
BP	71.41 ± 19.46	81.43 ± 16.98	80.62 ± 20.04	**84.91 ± 16.72**	79.20 ± 19.34	0.76
GH	81.67 ± 11.49	**83.09 ± 11.88**	78.28 ± 17.51	79.65 ± 14.26	80.06 ± 14.81	0.60
VT	75.21± 11.71	**79.05 ± 13.19**	75.60 ± 14.46	71.90 ± 15.83	75.27 ± 13.94	0.44
SF	59.61 ± 17.78	64.28 ± 20.65	65.23 ± 16.59	**66.38 ± 17.39**	63.89 ± 17.65	0.45
RE	49.57 ± 42.49	57.14 ± 48.49	55.21 ± 43.33	**57.47 ± 42.63**	54.47 ± 43.38	0.84
MH	**83.10 ± 11.98**	82.09 ± 16.23	80.11 ± 14.86	80.55 ± 14.76	81.23 ± 14.28	0.65
Total	65.93 ± 15.13	**72.43 ± 15.41**	70.59 ± 15.08	67.82 ± 17.08	69.13 ± 15.55	0.90
**KDQOL-36**						
PCS	65.64 ± 24.23	**79.28 ± 16.30**	69.37 ± 24.90	74.83 ± 22.58	70.82 ± 23.52	0.66
MCS	70.88 ± 17.16	**72.35 ± 21.16**	71.13 ± 20.90	71.72 ± 9.46	71.34 ± 19.59	0.72
BKD	50.64 ± 26.08	51.78 ± 29.19	54.59 ± 25.47	**55.82 ± 22.90**	53.43 ± 25.53	0.83
SPKD	84.91 ± 16.43	**90.80 ± 15.25**	89.13 ± 14.40	82.68 ± 18.56	87.06 ± 16.00	0.92
EKD	48.02 ± 28.85	44.79 ± 27.64	**49.61 ± 32.34**	43.21 ± 27.04	47.32 ± 29.72	0.93
Total	67.00 ± 13.26	**70.49 ± 14.20**	69.73 ± 14.06	67.27 ± 13.67	68.67 ± 13.75	0.88

*PF, Physical Functioning; RP, Limitations in Daily Role Functioning due to Physical Problems; BP, Bodily Pain; GH, General Health; VT, Vitality; SF, Social Functioning; RE, Limitations in Daily Role Functioning due to Emotional Problems; MH, Mental Health; PCS, Physical Component Summary; MCS, Mental Component Summary; BKD, Burden of Kidney Disease; SPKD, Symptoms and Problems of Kidney Disease; EKD, Effects of Kidney Disease. Period after kidney transplant having highest component score was given in bold*.

When measuring with the KDQOL-36, the overall mean score was 68.67 ± 13.75 and was the highest in the SPKD (87.06 ± 16.00). Two dimensions of the BKD and EKD had the lowest mean scores of all with 53.43 ± 25.53 and 47.32 ± 29.72, respectively. HRQOL domain scores measured by KDQOL-36 score were mainly high at 6 months to less than 1 year after KT. The BKD score has increased over time, reflective of a lower perceived BKD (Table 2).

### Validity and Reliability of the SF-36 and KDQOL-36

As indicated in Table 1, both the tools showed good reliability when used in our patients after KT. The reliability of the SF-36 was high with Cronbach's coefficients α = 0.90. Internal consistency in all domains was adequate (Cronbach's α ≥ 0.60), except for vitality (VT) (Cronbach's α = 0.44) and social activities (Cronbach α = 0.45). The reliability of the KDQOL-36 scale was overall high with Cronbach's coefficients α = 0.88, while Cronbach's α in domains ranged from 0.66 to 0.93.

**Figure 2:**
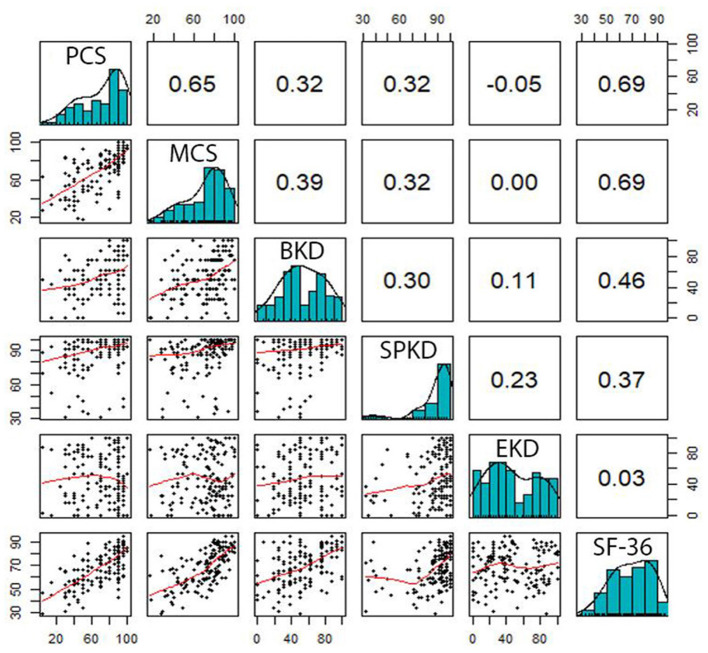
Correlation between the domains measured by the SF-36 and KDQOL-36. PCS, Physical Component Summary; MCS, Mental Component Summary; BKD, Burden of Kidney Disease; SPKD, Symptoms and Problems of Kidney Disease; EKD, Effects of Kidney Disease.

**Figure 3:**
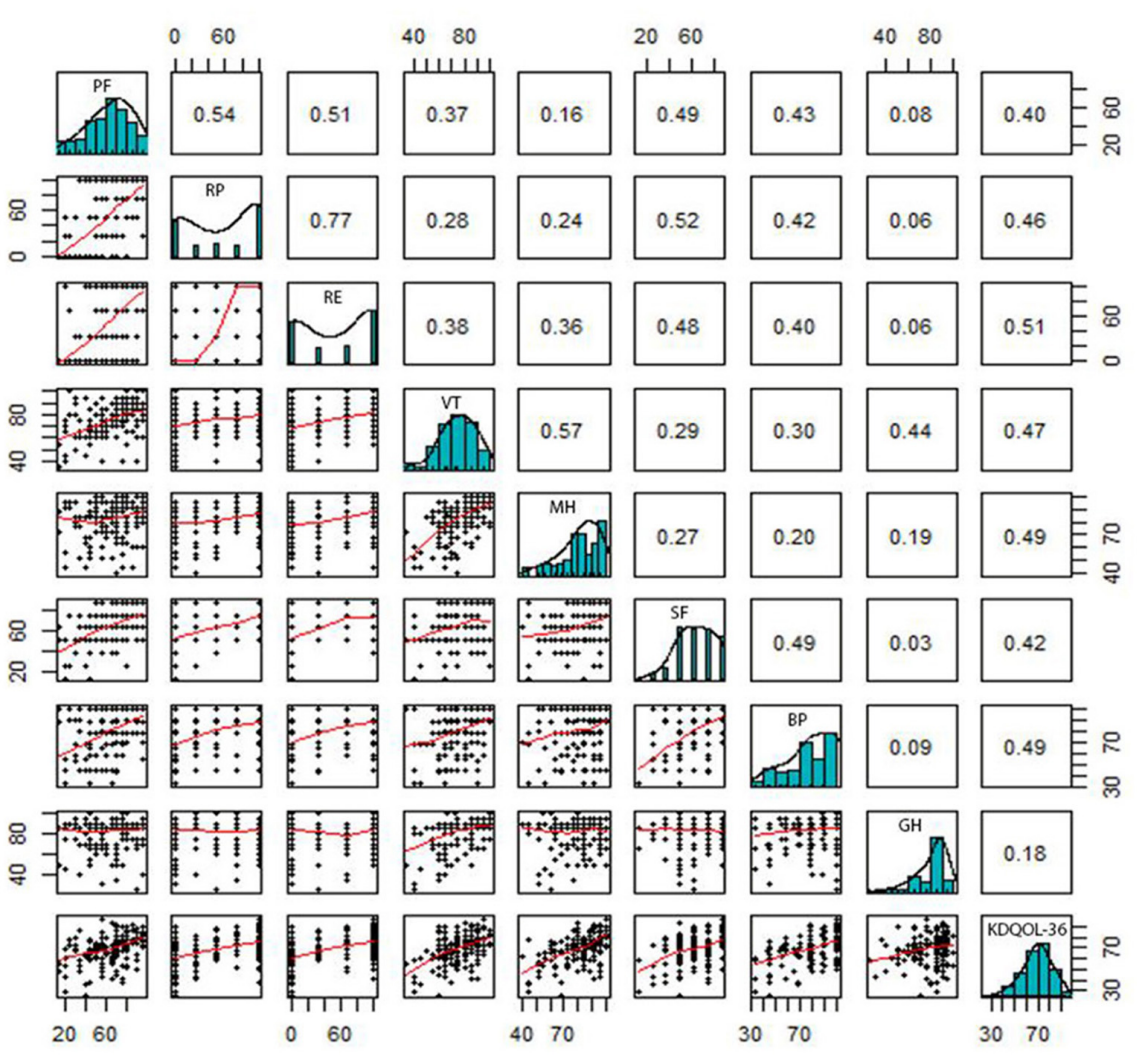
Correlation between the domains measured by KDQOL-36 and SF-36. PF, Physical Functioning; RP, Limitations in Daily Role Functioning due to Physical Problems; BP, Bodily Pain; GH, General Health; VT, Vitality; SF, Social Functioning; RE, Limitations in Daily Role Functioning due to Emotional Problems; MH, Mental Health.

Correlations between the domains of SF-36 and KDQOL-36 are pointed out in Figures 2, 3. In general, there were positive relationships between the dimensions assessed by the KDQOL-36 and SF-36 (correlation coefficient: 0.03–0.69). Similarly, the domains of the SF-36 also had positive correlations with KDQOL-36 (correlation coefficient: 0.18–0.51).

**Figure 4:**
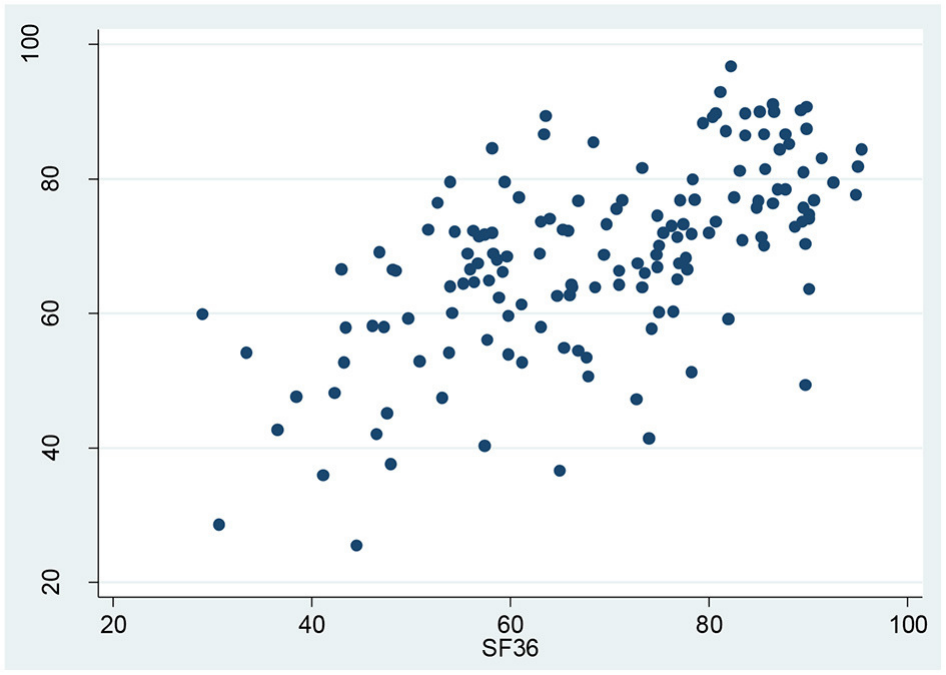
Correlation between the overall health-related quality of life (HRQOL) scores of the KDQOL-36 and SF-36.

The correlation coefficient between overall HRQOL scores of SF-36 and KDQOL-36 was 0.62, indicating a strong correlation between SF-36 and KDQOL-36. The comprehensive distribution of the correlation between the SF-36 and the KDQOL-36 is illustrated in Figure 4.

## Discussion

In addition to helping the patients in achieving optimal health compared to before the onset of disease, the patients after transplantation also need to ensure the balance between the function of organ transplant and the psychological integrity and physical integrity. This study highlights the importance of assessing the HRQOL in the KT patient after treatment, thereby determining the effectiveness of the instruments for the assessment of HRQOL in KT patients.

The study shows that the HRQOL of patients after KT fluctuated above average, both physical and mental health. The scores in each dimension changed according to the follow-up timelines within 3 years after transplantation but did not show a clear trend of change. The average scores generally obtained from the SF-36 and KDQOL-36 were quite similar (69.13 ± 15.55 compared to 68.67 ± 13.75). This is consistent with the recent report of Cordeiro in Brazil on 222 individuals after renal transplantation showing the average score by SF-36 and KDQOL of 64.7 ±24.3 and 78.5 ±16.8, respectively (13). An early report by Humar in 2,746 post-KT patients in the United States recorded higher scores with all domains above 60 points, highest in two health aspects, namely PF and RP (>90 points) (14). Compared with the individuals with ESKD who receive dialysis treatment in several previous studies in Vietnam, the overall HRQOL scores were 40.78 points in 112 patients at 103 Military Hospital and 48.89 points in 360 patients at People's Hospital 115 (9). Compared to the scores of physical and psychosocial domains of HRQOL in the previous reports (15, 16), there was a significant increase after transplant among most domains in our study. Though HRQOL of KT patients in Vietnam is not as high as in developed countries, the current result has improved significantly as compared to the patients with end-stage renal disease on hemodialysis. Among various alternative treatments for chronic kidney failure, most studies agree that KT significantly improves the HRQOL for patients (17–19).

When being assessed physically and mentally, patients reported that their HRQOL was not significantly affected, with average scores hovering over 50 points in all relevant domains. Meanwhile, when examining the effects of kidney disease using the KDQOL-36 specific tool, we obtained a relatively low score for the EKD (<50 points during follow-up), while BKD was reported to be 53.43 ± 25.53, with a high domain score of SPKD (87.06 ± 16.00). The findings presented in this study support the conclusion that the present treatment was capable of improving completely a number of symptoms and problems of kidney disease; however, the patient still finds it difficult to adapt their lives to new conditions partially due to monthly examination and lifelong antirejection medications. The previous studies reported that the specific domain scores of kidney disease were quite high, 78.5 in BKD, 81.7 in SPKD, and 83.8 in EKD (20), which were better scores as compared to this study. The differences between the studies may be mainly explained by a resource-scarce condition in clinical practice, economic hardship on the patient and their family, and aspects related to trust and belief. The current evidence supported the point that the conditions to support and care for post-KT Vietnamese patients are still not optimal as compared to the developed countries.

In the KDQOL-36 scale, the average total score of 12 questions on PCS and MCS, questions were shortened from the SF-36, quite similar to the SF-36 score. The present analysis showed that two questionnaires of the SF-36 and KDQOL-36 are highly reliable (Cronbach's α: 0.90 and 0.88, respectively) with a moderate positive relationship (correlation coefficient = 0.62), which showed that both scales giving the HRQOL scores were fairly comparable. The SF-36 questionnaire has been proven in many different healthcare settings, with various patient populations, so it is very effective for general HRQOL assessment and HRQOL comparison among multiple groups of subjects. However, it can be seen that the contents of the SF-36 scale do not help in making judgments about specific aspects in a particular population such as chronic kidney disease in this cohort, while 24 concentrated questions of the kidney disease known as the KDQOL-36 scale enable us to give a more accurate view. Disease-specific questionnaires have the advantage of focusing more on relevant aspects with high sensitivity in the changes in the clinical condition of that disease over time, thereby it is more practical for both physicians and patients (17). To our knowledge, there have been no identified questionnaires yet that can optimally measure all aspects of concern, while the questionnaires measuring HRQOL also can give mixed results once using on the same group of subjects (4). To our knowledge, it is not yet clear which instruments can measure all aspects of concern, while the instruments of HRQOL when used on the same patient group still commonly give different results (4). Current findings suggest that to make an HRQOL assessment of an individual after KT, it is possible to use either the SF-36 or the KDQOL-36 instruments to give the same results, but the results from this study also implied the need of depending on the specific aspect of HRQOL that the researcher care to choose the right instrument.

The results of this study should be interpreted within the context of some limitations. First our study is not powered to demonstrate the significance of two instruments in kidney disease-specific HRQOL domains. Second although the study did not evaluate the changes in HRQOL over time after KT vs. pre-KT, these were also valuable reference results to initially evaluate and determine the aspects of HRQOL in Vietnamese patients after KT. Future longitudinal studies are necessary to more comprehensively evaluate the process of HRQOL changes and their associated factors in the patients before and after KT, thereby improving the quality of treatment and increasing the ability in the detection of specific symptoms and subtle changes in the quality of life. Finally, the translated Vietnamese version of the KDQOL-36 scale has not been evaluated by RAND, and its psychometric properties have not been confirmed in a representative sample of the population; therefore, a proper translation of the KDQOL-36 scale may not guarantee that the scale is reliable and valid in the population.

## Conclusions

We found that there were slight fluctuations in the HRQOL score in domains within 3-year follow-up after KT. The mean SF-36 score was consistent with the mean KDQOL-36 score. High reliability and strong correlation were documented between the two instruments of SF-36 and KDQOL-36. In the developing condition in the clinical practice of Vietnam, both the SF-36 and KDQOL-36 can be considered as suitable instruments for assessing the HRQOL in patients after KT but initially need to be selected based on specific aspects that the physicians want to learn.

## Data Availability Statement

The raw data supporting the conclusions of this article will be made available by the authors, without undue reservation.

## Ethics Statement

The studies involving human participants were reviewed and approved by Ethics Board of the Hanoi Medical University. The patients/participants provided their written informed consent to participate in this study.

## Author Contributions

LV, NN, and TT have all made substantial contributions to conception and design of the study. TP and TT were responsible for the acquisition of data. LV, KV, and H-LV were responsible for analysis and interpretation of data. All authors were involved in drafting the manuscript or revising it critically for important intellectual content. All authors have given final approval of the version to be submitted.

## Conflict of Interest

The authors declare that the research was conducted in the absence of any commercial or financial relationships that could be construed as a potential conflict of interest.

## Publisher's Note

All claims expressed in this article are solely those of the authors and do not necessarily represent those of their affiliated organizations, or those of the publisher, the editors and the reviewers. Any product that may be evaluated in this article, or claim that may be made by its manufacturer, is not guaranteed or endorsed by the publisher.
